# Multiple lesions of gastrointestinal tract invasion by monomorphic epitheliotropic intestinal T-cell lymphoma, accompanied by duodenal and intestinal enteropathy-like lesions and microscopic lymphocytic proctocolitis: a case series

**DOI:** 10.1186/s13000-016-0519-x

**Published:** 2016-07-25

**Authors:** Hideki Ishibashi, Satoshi Nimura, Yoshiyuki Kayashima, Yasushi Takamatsu, Kunihiko Aoyagi, Naohiko Harada, Masanori Kadowaki, Takihiko Kamio, Shotaro Sakisaka, Morishige Takeshita

**Affiliations:** 1 Department of Gastroenterology and Medicine, Faculty of Medicine, Fukuoka University, Fukuoka, 814-0180 Japan; 2 Department of Pathology, Faculty of Medicine, Fukuoka University, Fukuoka, 814-0180 Japan; 3 Division of Medical Oncology, Hematology and Infectious Diseases, Faculty of Medicine, Fukuoka University, Fukuoka, 814-0180 Japan; 4 Department of Gastroenterology, Clinical Research Institute, National Hospital Organization, Kyushu Medical Center, Fukuoka, 810-8563 Japan; 5 Department of Hematology, Clinical Research Institute, National Hospital Organization, Kyushu Medical Center, Fukuoka, 810-8563 Japan; 6 Department of Pathology, Saiseikai Kumamoto Hospital, Kumamoto, 861-4193 Japan

**Keywords:** Gastrointestinal T-cell lymphoma, Intraepithelial lymphocytes, Enteropathy, Microscopic proctocolitis

## Abstract

**Background:**

In East Asia, monomorphic epitheliotropic intestinal T-cell lymphoma (MEITL), previously known as type II enteropathy-associated T-cell lymphoma (EATL), occurs more frequently than type I EATL, and coeliac disease is rare.

**Case presentation:**

Here we present four cases of MEITL in Japanese patients, including the endoscopic and pathological findings of their duodenal and colorectal lesions. Tumor specimens obtained from duodenal, intestinal, and colorectal biopsies in all four patients showed a diffuse intramucosal infiltration of small to/or medium-sized lymphoma cells and numerous atypical intraepithelial lymphocytes (IELs). These cells were immunohistologically positive for CD103, CD3, CD7, CD8, CD56, and T-cell intracellular antigen-1. Upper and lower gastrointestinal and antegrade double-balloon endoscopy revealed foci of edematous mucosa, with or without villous atrophy, in the non-neoplastic mucosa. Histological studies demonstrated duodenal and intestinal enteropathy-like lesions as well as microscopic (lymphocytic) proctocolitis with increased CD3- and CD8-positive and CD56-negative T-IELs in all four patients. The clinicopathological findings of the non-neoplastic lesions were similar to those characteristic of coeliac disease, suggesting that variants of coeliac disease may be present in the prodromal lesions of MEITL.

**Conclusions:**

Our study supports the need for random gastrointestinal biopsies to determine tumor spread, the features of MEITL in the particular patients, and the presence of prodromal non-neoplastic lesions.

## Background

Enteropathy-associated T-cell lymphoma (EATL) is uncommon worldwide but occurs more frequently in areas with a high prevalence of coeliac disease, particularly in Northern Europe and America [1]. In the recent World Health Organization (WHO) classification, EATL is classified into two types. In Northern Europe and America, ~80 % of type I EATL cases consist of CD103- and CD30-positive, CD56- and CD8-negative large-cell lymphomas. These cases are closely associated with the occurrence of coeliac disease [2]. In patients with type II EATL, which is now referred to as monomorphic epitheliotropic intestinal T-cell lymphoma (MEITL), complications of coeliac disease are rare. Most MEITLs are CD103-, CD56-, and CD8-positive, CD30-negative monomorphic lymphomas with small- to medium-sized cells [3]. In East Asia, type I EATL is rare, consistent with the rarity of coeliac disease. The latter is characterized by an allergic reaction to gluten and an association with HLA-DQ 2(B1*02) and DQ8 serotypes [2, 4, 5]. MEITL is predominantly located in the small intestine but the background presence of enteropathy is controversial [3, 6]. In East Asia, MEITL typically lacks the persistent clinicopathological features of enteropathy, in contrast to a worldwide study in which a clinical history of coeliac disease was determined in four of 15 (27 %) patients with MEITL [7]. Compared with the general population, patients with coeliac disease have a 70-fold higher risk of microscopic (lymphocytic or collagenous) colitis accompanied by either an edematous or a normal-looking mucosa [8]. Here we provide a detailed report of the endoscopic and pathological findings of four Japanese patients with multiple lesions of gastrointestinal (GI) MEITL complicating the duodenal and intestinal enteropathy-like lesions and microscopic (lymphocytic) foci of proctocolitis characterized by increased numbers of intraepithelial lymphocytes (IELs) [8–10]. These cases demonstrate that variants of coeliac disease may be present in East Asian patients with MEITL and thus the necessity of a random whole-GI-tract biopsy in this group of MEITL patients.

## Case presentation

### Case selection and histology

The four selected cases were histologically classified according to the WHO system of classification [1], in which type II EATL is now referred to as MEITL. All four patients were seronegative for antibodies against human T-cell lymphotropic virus type 1 and none had Epstein–Barr virus (EBV) infection, as determined by in situ hybridization of EBV-encoded RNA (DakoCytomation, Glostrup, Denmark). Tumor stage was classified according to the modified Ann Arbor staging system [11]. Tissue specimens were fixed with 10 % formalin, embedded in paraffin, and stained with hematoxylin and eosin (H&E). In non-neoplastic mucosa, the detection of >30 small IELs per 100 epithelial cells was considered to indicate positive, specific findings. Scattered small IELs without irregular nuclei were presumed to be reactive. Small to/or medium-sized lymphocytes with irregular nuclei and densely infiltrating epithelial glands were defined as atypical IELs. Enteropathy-like lesions in the duodenum and small intestine were recognized based on the increased number of IELs and the presence of villous atrophy [8].

### Immunohistochemistry

Tumor immunohistology was evaluated by incubating formalin-fixed tumor samples with a panel of monoclonal and polyclonal antibodies, according to the ChemMate Envision (DakoCytomation) method. Peroxidase reactions were developed using diaminobenzidine as the substrate. Staining for CD20 (clone: L26; Nichirei, Tokyo, Japan), CD3 (PS1; Leica, Newcastle, UK), T-cell receptor (TCR)-βF1 (8A3; Endogen, Rockford, IL, USA), TCRCγM1 (γ3.20; Endogen), CD4 (4B12, Leica), CD5 (4C7, Leica), CD7 (LP15, Leica), CD8 (C81/44B; Leica), CD30 (BerH2; DakoCytomation), CD103 (EPR4166 [2]; Abcam, Cambridge, UK), CD56 (1B6; Leica), and T-cell intracellular antigen (TIA1, 2GP; Immunotech, Marseille, France) was performed after antigen retrieval. Samples in which >25 % of the cells were labeled with a particular antibody marker were classified as positive for that marker.

The clinical and pathological features of the four patients diagnosed with MEITL are summarized in Tables 1 and 2.

**Table 1:** Clinical features, treatments and prognosis in four cases of MEITL

	Case 1	Case 2	Case 3	Case 4
Age (years)	60	40	50	70
Sex	M	F	F	M
Chief complaints	Diarrhea	Diarrhea	Abdominal distention	Nausea
Duration of chief complains (mons)	17	3	1	2
Total protein (g/dl)	4	4.4	6.6	6.4
Albumin (g/dl)	2.1	2.9	3.8	3
LDH (IU/l)	165	156	187	150
sIL2R (U/ml)	963	1419	4150	587
Anti-gliadin/-transglutaminase tests	−/−	ne	ne	ne
*Helicobacter pylori* IgG antibody	−	ne	−	+
Gastrointestinal perforation	−	+ (after treatment)	−	−
Bone marrow tumour invasion	+ (30 %)	−	+ (30 %)	+ (20 %)
Clinical stage	IV	I	IV	IV
Treatment (regimen)	CHASE, SCT	THP-COP, surgery	CHOP, SCT	SMILE
Response	Partial response	No response	Partial response	Partial response
Survival times (months)	36, dead	12, dead	9, dead	9, dead

*CHASE* Cyclophosphamide, cytarabine, etoposide, dexametahsone, *SCT* Stem cell transplantation, *THP-COP* Pirarubicin, cyclophosphamide, vincristine, prednisolone, *CHOP* Cyclophosphamide, doxorubicin, vincristine, prednisolone, *SMILE* Dexamethasone, methotrexate, ifosfamide, L-asparaginase, etoposide; *ne* not examined

**Table 2:** Endoscopic and histological findings in the GI tract and cell-surface markers in four patients with MEITL

	Case 1	Case 2	Case 3	Case 4
Duodenal tumour lesions	Edematous mucosa	Edematous mucosa	Reddish granular mucosa	Submucosal tumors, pinhole-like stenosis
Intestinal tumour lesions	Diffuse thickening, circumferential ulcers	Diffuse thickening, ulcerative tumours	Diffuse thickening	Diffuse thickening
Colonic tumor lesions	Edematous mucosa	Edematous mucosa	Edematous mucosa with erosions and ulcers	Edematous mucosa
Duodenal nonneoplastic lesion	Edematous mucosa, villous atrophy	Edematous mucosa, villous atrophy	Edematous mucosa, villous atrophy	Edematous mucosa, villous atrophy
Intestinal nonneoplastic lesion	Diffuse edematous mucosa, villous atrophy	Diffuse granular mucosa, villous atrophy	Diffuse granular mucosa, villous atrophy	Diffuse granular mucosa, villous atrophy
Colorectal nonneoplastic lesion	Edematous mucosa	Edematous mucosa	Edematous mucosa	Edematous mucosa
Tumour invasion (S/D/I/C/R)	+/+/+/+ (cecum)/−	−/+/+/+ (cecum) /−	ne/+/+/+/+	ne/+/+/+/+
Duodenal and intestinal enteropathy	+	+	+	+
Lymphocytic proctocolitis	+	+	+	+
Increase of reactive T-IELs (D/I/C/R)	+/+/+/+	+/+/+/−	+/+/+/+	+/+/+/+
CD103	+	+	+	+
TCR βF1	+	−	+	−
TCR CγM1	−	+	−	+
CD3	+	+	+	+
CD4	−	−	−	−
CD7	+	+	+	+
CD8	+	+	+	+
CD56	+	+	+	+
TIA1	+	+	+	+
CD5	−	−	−	−
CD30	−	−	−	−

*S* Stomach, *D* Duodenum, *I* Intestine, *C* Colon, *R* Rectum, *IELs* Intraepithelial lymphocytes, *TCR* T-cell receptor, *TIA* T-cell intracellular antigen

#### Case 1

**Fig. 1:**
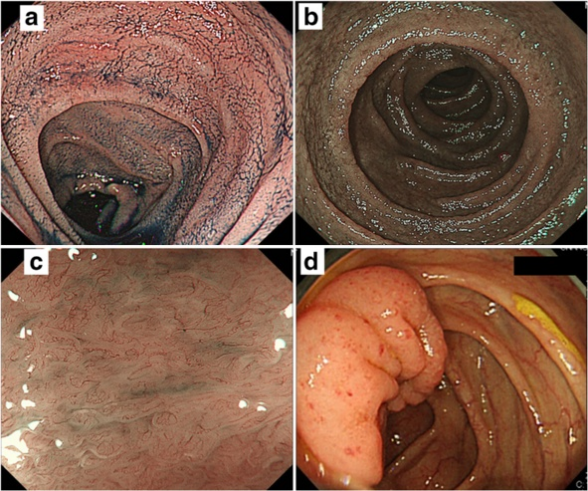
Case 1 (**a**–**b**). **a**: Indigo carmine spray enhancement and **b**: narrow-band imaging (NBI) of the duodenal second portion shows the edematous mucosa with villous atrophy. **c**: Magnifying endoscopic view with NBI of the duodenal second portion shows cerebriform or flat patterns with villous atrophy. **d**: Lower gastrointestinal (GI) endoscopic view of the ileocecal valves shows the edematous mucosa with small erosions; normal-looking mucosa is seen in the ascending colon

**Fig. 2:**
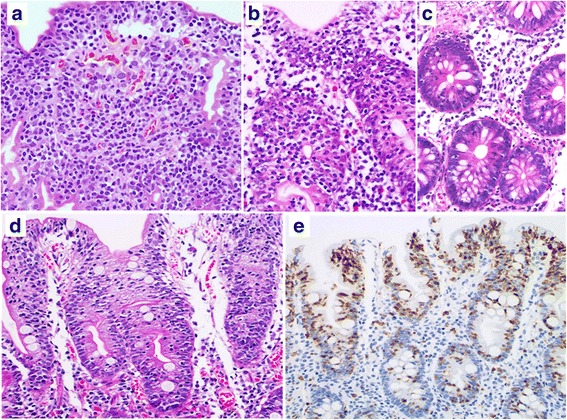
Patient 1 (**a**–**c**). **a**: A diffuse infiltrate of small to medium-sized atypical lymphocytes and many atypical IELs are seen in the duodenal (**a**) and cecal (**b**) mucosa. **c**: Small intraepithelial lymphocytes are scattered in the non-neoplastic ascending colonic mucosa, indicating lymphocytic colitis. Patient 2 (**d**, **e**). **d**: Duodenal enteropathy with atrophic villi and increased IELs. **e**: Many small CD8-positive IELs are seen in the duodenum (**a**, **b**, **c**, **d**: H&E stain; **e**: immunohistochemistry, hematoxylin stain)

A 60-year-old man who was admitted with persistent diarrhea and a 10 kg weight loss in 17 months. After a tentative diagnosis of coeliac disease he was followed in another hospital for 8 months. His laboratory data revealed hypoproteinemia and elevated soluble interleukin-2 receptor (sIL2R). Anti-gliadin and anti-transglutaminase antibodies, serum-specific markers for coeliac disease, were negative [12]. Abdominal computed tomography (CT) revealed thickening of the jejunal and ileal walls and mild dilatation. On upper GI endoscopy, a reddish depressed lesion with erosion was seen in the gastric body; in the second portion of the duodenum, the edematous mucosa was accompanied by villous atrophy (Fig. 1a). Upper GI endoscopy with narrow-band imaging (NBI) showed an edematous mucosa, while magnifying endoscopy with NBI revealed a cerebriform or flat pattern with villous atrophy in the second duodenal portion (Fig. 1b, c). Capsule endoscopy demonstrated a diffuse edematous and granular mucosa with villous atrophy and circumferential ulcers in the jejunum and ileum. On lower GI endoscopy, an edematous mucosa with small erosions at the ileocecal valve were present (Fig. 1d) whereas the findings along the whole colorectal mucosa were nearly normal. Biopsy specimens from the gastric, duodenal, jejunal, and cecal mucosae showed diffuse intramucosal invasion by small to medium-sized atypical lymphocytes with irregular hyperchromatic nuclei, in addition to many atypical IELs (Fig. 2a, b). Immunohistologically, the atypical lymphocytes were positive for CD103, TCR-βF1, CD3, CD7, CD8, CD56, and TIA1. Other biopsy specimens from the duodenal second portion showed villous atrophy and chronic inflammatory infiltrates. The T-IELs comprising the infiltrates were positive for CD103, CD3, CD7, CD8, and TIA1 but negative for CD56 and CD5. Evaluation of the colorectum showed colitis with an increased number of T-IELs, all of which were of the same phenotype (Fig. 2c). A bone marrow biopsy revealed scattered infiltrates of CD103-, CD3-, CD8-, CD56-positive and CD5-negative atypical lymphocytes; these accounted for ~30 % of the nucleated cells. The patient was treated with a chemotherapy regimen of CHASE (cyclophosphamide, cytarabine, etoposide, and dexamethasone) and underwent autologous stem cell transplantation. Despite a partial response to chemotherapy, he died of sepsis 3 years after disease onset.

#### Case 2

**Fig. 3:**
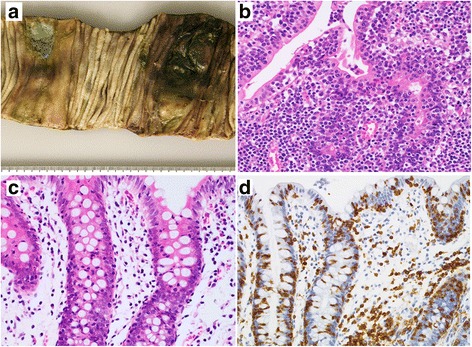
Patient 2 (**a**–**d**). **a**: Two circumferentially ulcerated tumors are evident on gross examination of the resected jejunum. Thickening of Kerckring’s folds and granular changes in the mucosal surface are seen in the mucosa surrounding the tumor. **b**: Histological findings of the jejunum include a diffuse infiltrate of medium-sized atypical lymphocytes with round nuclei in the crypt epithelium and lamina propria of the peripheral zone of the jejunal tumor. **c**: Increased small IELs are present in the ascending colon (**c**); the CD8 positivity of the IELs (**d**) is indicative of lymphocytic colitis. (**b**, **c**: H&E stain, **d**: immunohistochemistry, hematoxylin stain)

A 40-year-old woman who was admitted with diarrhea and a weight loss of 6 kg in 3 months. Her laboratory data revealed hypoproteinemia and an elevated sIL2R level. On upper GI endoscopy, the gastric mucosa was nearly normal in its appearance whereas the mucosa of the duodenal second portion was edematous. Antegrade double-balloon endoscopy of the duodenal third portion and the jejunum showed a diffuse edematous mucosa, erosion, and ulcerative tumors in both. On lower GI endoscopy, an edematous mucosa and reddish polypoid lesions were seen in the terminal ileum together with an edematous colorectal mucosa. Biopsy specimens from the duodenal third portion, intestine, and colon showed a diffuse, intramucosal infiltrate of medium-sized atypical lymphocytes with coarse chromatin together with many atypical IELs. Other biopsy specimens from the duodenal second portion (Fig. 2d, e) and intestine revealed chronic inflammatory changes with villous atrophy and an abundance of CD103-, CD8-positive, CD56-negative T-IELs. Scattered infiltrates of CD3-, CD4-, and CD8-positive, CD103- and CD56-negative lymphocytes were identified on bone marrow specimens but there was no evidence of bone marrow invasion. She was treated with a chemotherapy regimen of THP-COP (pirarubicin, cyclophosphamide, vincristine and prednisolone) but 10 months after the initial diagnosis was readmitted to our hospital with acute abdominal pain due to intestinal perforation. A partial jejunal resection revealed multiple and circumferential ulcerated tumors with or without perforation; the mucosa surrounding the tumors was characterized by fold thickening with granular changes (Fig. 3a). Histologically, a diffuse infiltrate of medium-sized atypical lymphocytes accompanied by atypical IELs was present in the mucosa, in the peripheral zones of the tumors (Fig. 3b). Enteropathy-like lesions with villous atrophy and an abundance of IELs were evident in the mucosal layer outside the main tumors. Preoperative biopsy specimens obtained from the ascending colon to the rectum revealed colitis, with increased CD3- and CD8-positive and CD56-negative T-IELs (Fig. 3c, d). She died of the disease 2 months after surgery.

#### Case 3

**Fig. 4:**
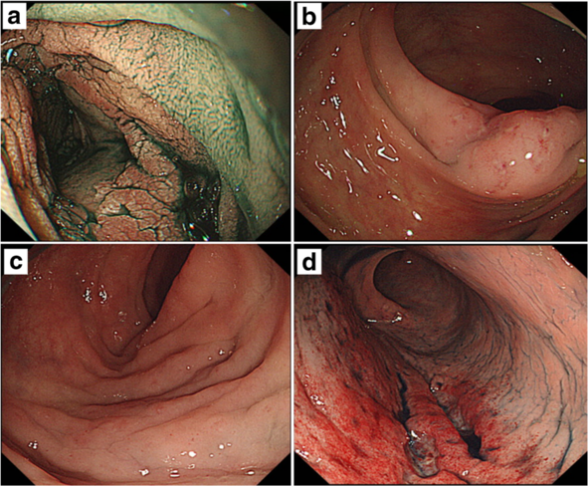
Patient 3 (**a**–**d**). **a**: Upper GI endoscopic view of the duodenal second portion shows an edematous mucosa with a nodular or mosaic pattern. **b**: A lower GI endoscopic view of the ascending colon shows an edematous mucosa with multiple erosions. Lower GI endoscopic views show an edematous mucosa in the sigmoid colon (**c**) and reddish longitudinal ulcers in the rectum (**d**)

**Fig. 5:**
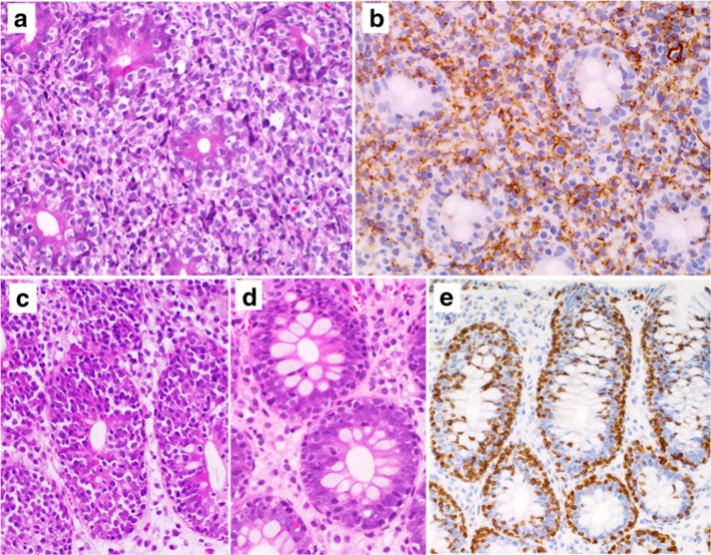
Patient 3 (**a**, **b**). **a**: Tumors of the sigmoid colon are characterized by a diffuse infiltrate of atypical medium-sized lymphocytes and increased atypical IELs. **b**: Infiltrating atypical lymphocytes are diffusely positive for CD56. Patient 4. **c**: A prominent increase in the number of small atypical IELs is seen in the cecal mucosa. **d**: Many small IELs are present in the descending colon. **e**: Reactive infiltrating IELs of the colon are positive for CD3 (**a**, **c**, **d**: H&E stain, **b**, **e**: immunohistochemistry, hematoxylin stain)

A 50-year-old woman who was admitted to our hospital for abdominal distension. Her laboratory data revealed an elevated sIL2R. On abdominal CT, long segmental thickening of the jejunal and ileal wall with dilatation was evident. Upper GI endoscopy revealed an edematous and reddish granular mucosa with white villi in the duodenal second portion (Fig. 4a). On lower GI endoscopy, a diffuse granular mucosa with villous atrophy in the terminal ileum, an edematous mucosa with multiple erosions in the ascending and sigmoid colon, and reddish longitudinal ulcers in the rectum were seen (Fig. 4b–d). Biopsy specimens from the duodenal second portion, intestine, and colorectum revealed a diffuse intramucosal infiltrate of medium-sized atypical lymphocytes with many atypical IELs (Fig. 5a, b). Other biopsy specimens from the duodenum, jejunum, and colorectum indicated chronic inflammatory changes of the propria mucosae and increased CD3- and CD8-positive, CD56-negative T-IELs. An invasion by CD3-, CD8-, and CD56-positive atypical lymphocytes was seen on the bone marrow specimens. She was treated with cyclophosphamide, doxorubicin, vincristine, and prednisolone and high-dose methotrexate/cytarabine followed by allogeneic stem cell transplantation. Despite a partial response to chemotherapy, she died 9 months after disease onset.

#### Case 4

A 70-year-old man admitted for nausea. His laboratory data revealed pancytopenia and hypoproteinemia. *Helicobacter pylori* IgG antibody was positive in serum. Abdominal CT showed a 30-mm-diameter tumor in the duodenal second portion and thickening of the jejunal wall. On ^18^F-fluorodeoxyglucose (FDG) positron emission tomography, there was FDG uptake in the duodenal second portion. Upper GI endoscopy revealed pinhole-like severe stenosis, a submucosal tumor at the anal side of the stenosis, and edematous mucosa in the duodenal second portion. Lower GI endoscopy showed a granular mucosa of the terminal ileum and edematous or normal-looking mucosa in the colorectal mucosa. Duodenitis with villous atrophy and abundant IELs was evident on biopsy specimens from the duodenal second portion taken outside the tumor. The tumor itself consisted of small to medium-sized atypical lymphocytes both among the epithelial cells and in the mucosal layer. The cecal mucosa was characterized by a severe infiltrate of atypical IELs (Fig. 5c). Other biopsy specimens from the duodenum, descending colon, and rectum showed chronic inflammatory changes with increased CD3- and CD8-positive, CD56-negative T-IELs (Fig. 5d, e). Infiltrating atypical lymphocytes of the bone marrow were positive for CD3, CD8, CD103, and TCRCγM1, but negative for CD56. He was treated with a chemotherapy regimen of SMILE (dexamethasone, methotrexate, ifosfamide, L-asparaginase and etoposide). After chemotherapy, abdominal CT revealed a reduction of the tumor. Despite a partial response to chemotherapy, he died of sepsis 9 months after disease onset.

## Discussion

There are several reports in the literature describing gastroduodenal and colorectal tumors of MEITL and type I EATL, both in European and in East Asian patients [3, 6, 13, 14]. In the latter group, gastric, duodenal, and colorectal MEITL tumors were reported in three (12 %), eight (31 %), and six (23 %) of the 26 cases of MEITL, respectively [13]. In their study of European patients, Schmitt-Graff et al. reported tumorous lesions in six of 20 cases of MEITL (30 %) [14]. In our study, patient 1 had depressed lesions and an edematous mucosa, with tumor cells in the stomach, duodenum, and colorectum. Patients 2 and 4 had ulcerative or submucosal tumors in the duodenum and an edematous mucosa with tumor cells in the colorectum. Patient 3 had diffusely granular mucosal lesions, with tumor cells in the duodenum and multiple ulcerated colorectal tumors. Thus, our study demonstrates that MEITL can expand discontinuously into the mucosa along the entire course of the GI tract.

The endoscopic findings in 12 of the 15 reported cases of MEITL included circumferential ulcerated tumors and an edematous and granular mucosa, with a nodular or mosaic pattern, in the duodenum, jejunum, and ileum (Table 3) [15–24]. In addition, in all four patients, the non-neoplastic lesions consisted of an edematous or granular mucosa, with or without villous atrophy, in the duodenum and intestine. In patient 1, a cerebriform or flat mucosal pattern with villous atrophy was seen in the duodenum on magnifying endoscopy with NBI. These findings are typical of coeliac disease [25]. Endoscopically, our study shows that an edematous and granular mucosa, with or without villous atrophy, in the duodenum and intestine is characteristic of the non-neoplastic and prodromal lesions of MEITL.

**Table 3:** Endoscopic findings of duodenum, intestine and colorectum in reported cases of MEITL

Case no.	Age/gender	Locations and endoscopic findings	References
1	77/M	Duodenum: Edematous mucosa	[14]
		Jejunum: Mass with circumferential ulcers	
2	56/M	Sigmoid colon: Edematous mucosa, multiple discrete ulcers	[14]
		Rectum: Edematous mucosa, multiple discrete ulcers	
3	63/F	Ileum: Edematous mucosa	[15]
		Appendix: Submucosal tumor-like mass	
4	52/F	Jejunum: Edematous mucosa	[16]
		Ileum: Shallow circumferential ulcers	
5	65/M	Jejunum: Widespread white granular villi	[17]
6	70/M	Jejunum: Edematous mucosa, fusion on villi, multiple small shallow ulcers	[18]
7	60/M	Duodenum: Fine granular patterns with ulcers	[19]
		Jejunum: Fine granular patterns with ulcers	
		Ileum: Edematous mucosa, circumferential ulcers	
8	16/M	Ileocecum: Multifocal irregular ulcers	[20]
9	62/M	Duodenum: Huge ulcer	[20]
10	54/M	Jejunum; Multiple discrete ulcers	[21]
11	71/M	Ascending colon: Huge ulcerated tumor	[22]
		Left side colon: Ordinary-looking mucosa	
12	67/M	Cecum: Hyperemic, thickened mucosa with central ulcer	[23]
		Descending colon: Fresh-like flat thickened lesion	
13	50/M	Ileum: Circumferential shallow ulcers	[23]
		Ascending colon: An ulcerative lesion with hyperemic edematous mucosa	
14	48/M	Jejunum: Diffuse mucosal thickening and nodularity with multiple shallow ulcers	[23]
15	55/F	Jejunum: Encircling ulcer, edematous mucosa with innumerable fine granular elevations and shallow ulcers	[23]

*M* Male, *F* Female

Microscopic colitis consists of lymphocytic and collagenous colitis with >20 IELs per 100 surface epithelial cells and no or little architectural distortion of the crypts [10]. While of uncertain etiology, both lymphocytic and collagenous microscopic colitis are occasionally seen in patients with coeliac disease, hypothyroidism, diabetes mellitus, and rheumatoid arthritis [26]. In three reported cases of colonic MEITL, ulcerated tumors and hyperemic as well as normal-looking mucosa outside the tumors were detected (cases 2, 11, and 12 in Table 3) [15, 23, 24]. However, only the patient described by Kim et al. had colonic MEITL characterized by presumably normal mucosa with tumor cell invasion [23]. All four of our MEITL patients had an edematous and presumably normal colorectal mucosa, with an increased number of IELs, and without tumor cells outside the tumors. If lymphocytic proctocolitis is detected it must be distinguished from prodromal lesions of MEITL, even in East Asian patients. This study supports the importance of performing a random biopsy of the whole GI tract to detect the spread of MEITL and to identify the underlying non-neoplastic disorders.

Duodenitis, enteritis, and lymphocytic proctocolitis with increased T-IELs were features of all four cases described herein. In addition, the T-IELs were positive for CD103, CD3, CD7, and CD8 and negative for CD56 and CD5. These findings are similar to those of coeliac disease [9, 27]. In East Asia, rather than type II EATL, these findings were referred to as MEITL, because of the absence of background histological findings of enteropathy [3]. However, persistent diarrhea and weight loss have been reported in Asian patients with MEITL, and histologically confirmed enteropathy-like lesions were detected in eight of 69 cases of intestinal T/NK-cell lymphoma (12 %), including seven cases of MEITL [28]. Kikuma et al. [13] also reported the presence of some degree of enteropathy-like lesions in 11 of 22 MEITL patients (50 %). Coeliac disease characterized by anti-gliadin and anti-transglutaminase antibodies is rare in East Asia, whereas based on array comparative genomic hybridization, six of eight (75 %) Asian patients with MEITL had a gain of chromosome 9q34, as is frequently found in refractory coeliac disease and type I EATL [2, 29]. Thus, variants of coeliac disease may play a role in inducing MEITL. However, refractory coeliac disease, suggesting of prodromal lesion of type I EATL, frequently has CD8-negative and occasionally CD30-positive T-IELs in duodenum and intestine [9]. Further, Narismägi et al. [30] demonstrated that mutations of STAT5, JAK3, and G-protein-coupled receptor are common features of MEITL. Thus, type I EATL and MEITL may include a variety of oncogenes in lymphomagenesis. It is necessary to find etiological factors in patients with variant of coeliac disease and MEITL.

## Conclusions

Duodenal and intestinal enteropathy-like lesions and microscopic (lymphocytic) proctocolitis with increased T-IELs resemble the features of coeliac disease but may be prodromal lesions of MEITL. Moreover, variants of coeliac disease may be present in MEITL. Random biopsies are necessary to determine the occurrence of tumor spread as well as the characteristics of MEITL and prodromal non-neoplastic lesions.

## Abbreviations

CT: computed tomography; EATL: enteropathy-associated T-cell lymphoma; FDG: fluorodeoxyglucose; GI: gastrointestinal; IELs: intraepithelial lymphocytes; JAK: Janus kinase; MEITL: monomorphic epitheliotropic intestinal T-cell lymphoma; NBI: narrow-band imaging; STAT: signal transducers and activator of transcription; TCR: T-cell receptor; TIA1: T-cell intracellular antigen 1; WHO: World Health Organization
